# Fine mapping of the *BnaC04.BIL1* gene controlling plant height in *Brassica napus* L

**DOI:** 10.1186/s12870-021-03137-9

**Published:** 2021-08-05

**Authors:** Mao Yang, Jianbo He, Shubei Wan, Weiyan Li, Wenjing Chen, Yangming Wang, Xiaomei Jiang, Pengfei Cheng, Pu Chu, Wenbiao Shen, Rongzhan Guan

**Affiliations:** 1 National Key Laboratory of Crop Genetics and Germplasm Enhancement, Jiangsu Collaborative Innovation Center for Modern Crop Production, Nanjing Agricultural University, Nanjing, 210095 China; 2 College of Life Sciences, Laboratory Center of Life Sciences, Nanjing Agricultural University, Nanjing, Jiangsu China

**Keywords:** *Brassica napus*; semi-dominant; dwarf; single nucleotide polymorphism; gene mapping

## Abstract

**Background:**

Plant height is an important architecture trait which is a fundamental yield-determining trait in crops. Variety with dwarf or semi-dwarf phenotype is a major objective in the breeding because dwarfing architecture can help to increase harvest index, increase planting density, enhance lodging resistance, and thus be suitable for mechanization harvest. Although some germplasm or genes associated with dwarfing plant type have been carried out. The molecular mechanisms underlying dwarfism in oilseed rape (*Brassica napus* L.) are poorly understood, restricting the progress of breeding dwarf varieties in this species. Here, we report a new dwarf mutant *Bndwarf2* from our *B. napus* germplasm. We studied its inheritance and mapped the dwarf locus *BnDWARF2*.

**Results:**

The inheritance analysis showed that the dwarfism phenotype was controlled by one semi-dominant gene, which was mapped in an interval of 787.88 kb on the C04 chromosome of *B. napus* by Illumina Brassica 60 K Bead Chip Array. To fine-map *BnDWARF2*, 318 simple sequence repeat (SSR) primers were designed to uniformly cover the mapping interval. Among them, 15 polymorphic primers that narrowed down the *BnDWARF2* locus to 34.62 kb were detected using a F_2:3_ family population with 889 individuals. Protein sequence analysis showed that only BnaC04.BIL1 (BnaC04g41660D) had two amino acid residues substitutions (Thr187Ser and Gln399His) between ZS11 and *Bndwarf2*, which encoding a GLYCOGEN SYNTHASE KINASE 3 (GSK3-like). The quantitative real-time PCR (qRT-PCR) analysis showed that the *BnaC04.BIL1* gene expressed in all tissues of oilseed rape. Subcellular localization experiment showed that BnaC04.BIL1 was localized in the nucleus in tobacco leaf cells. Genetic transformation experiments confirmed that the *BnaC04.BIL1* is responsible for the plant dwarf phenotype in the *Bndwarf2* mutants. Overexpression of *BnaC04.BIL1* reduced plant height, but also resulted in compact plant architecture.

**Conclusions:**

A dominant dwarfing gene, *BnaC04.BIL1*, encodes an GSK3-like that negatively regulates plant height, was mapped and isolated. Our identification of a distinct gene locus may help to improve lodging resistance in oilseed rape.

**Supplementary Information:**

The online version contains supplementary material available at 10.1186/s12870-021-03137-9.

## Background

Oilseed rape (*Brassica napus* L.) is one of the most important oil crops worldwide, and provides high-quality vegetable oil for human diets, protein-rich feed for animals, and raw materials for industrial processes. Variety with dwarf or semi-dwarf phenotype is a major objective in the breeding because dwarfing architecture can help to increase harvest index, increase planting density, enhance lodging resistance, and thus be suitable for mechanization harvest [1]. To find available germplasm or genes associated with dwarfing plant type for *B. napus* breeding, some efforts have been carried out. For example, the dwarfness-associated genes in *B. napus*, including *DS-1* [2], *ndf-1* [3], *DS-3* [4], *DS-4* [5], *G7* [6], *BnaDwf.C9* [7], have been positioned or identified. Additionally, the *Bndwf1* was fine-mapped on the A9 chromosome to a 152-kb interval [8]. However, the molecular mechanism(s) underlying the development of the dwarf phenotype in *B. napus* remain elusive. The lack of innovation on *B. napus* ideal type breeding is mainly due to absence of successfully applied cultivar in vast oilseed rape production region.

Dwarfism is usually related to plant hormone biosynthesis and signal transduction, such as auxin [9, 10], gibberellin (GA) [1, 11], and brassinosteroid (BR) [12, 13]. Auxin affects plant height by regulating cell division, elongation, and differentiation [14]. GA mainly affects the elongation of stem and internode to regulate plant height [1]. Relationship between plant dwarf stature and genes in auxin and GA biosynthesis and signal transduction pathways has been well-documented [4, 15–19]. Defects in BR biosynthesis and signaling pathways can lead to dwarfing phenotypes. During BR biosynthesis, many synthases belong to the cytochrome P450 monooxygenase (CYP) gene. Defects of these synthases can lead to dwarfing phenotypes. For instance, *CPD* (*constitutive photomorphogenesis and dwarfism*) encodes a steroid 23α-hydroxylase enzyme, a member of CYP90A family, which acts in the conversion of cathasterone to teasterone in the BR biosynthetic pathway [20]. The loss-of-function mutations of *AtCPD* gene leads to the dwarfing phenotype, when over-expression of *CPD* gene can restore the plant height and plant type [20]. *BRD1* (*BR-deficient dwarf 1*) gene encodes the final catalytic enzyme (BR-C6 oxidase) in BR biosynthesis, mutation of which cause dwarfing phenotype. Rice *brd1* was the first report to describe the phenotypic characterization of a BR-deficient mutant in monocot plants, and showed the phenotype of leaf sheath small, leaves wrinkled, internodes short, fewer tillers. The exogenous application of BL can restore the plant type of the *brd1* mutants [21]. The *dwf4* (*CYP90B1*) in *Arabidopsis*, *dwarf2* (*CYP90D*) and *dwarf11* (*CYP724B1*) in rice encode P450 monooxygenase to involve in BR biosynthesis, loss-of-function mutations of which reduce the endogenous BR levels and consequently confer reduced plant height [13, 22, 23]. The *Arabidopsis DET2* (*de-etiolated 2*) is a key gene in BR biosynthesis and allow an assignment for this steroid’s role in plant development [24, 25]. The *Arabidopsis det2* mutant [24] and the maize *na1* (*nana plant1*) mutant [26] were the loss-of-function of *DET2* gene lead to the dwarfing phenotype, dark green leaves, and have reduced fertility.

BR signal transduction is a signaling cascade from the BR receptor to the expression of BR target genes, which plays an important role in various developmental and growth processes in plants [27]. Researches during the past several decades have accumulated extensive knowledge of BR signaling pathways in model plants [28–30], such as *Arabidopsis* and rice. It is well documented that BRs are perceived extracellularly by the BR-INSENSITIVE1/BRI1-ASSOCIATED KINASE1 (BRI1-BAK1) [31–33] complex. Afterwards, the binding between BRs and BRI1-BAK1 complex could initiate signal transduction to BRASSINAZOLERESISTANT1/BRI1-EMS-SUPPRESSOR1 (BZR1/BES1) [34, 35] through CONSTITUTIVE DIFFERENTIAL GROWTH1 (CDG1) [36] and BR SIGNALING KINASE1 (BSK1) [37], then BRI-SUPPRESSOR1 (BSU1) [38], BRASSINOSTEROID INSENSITIVE2 (BIN2) [39, 40], as well somehow PROTEIN PHOSPHATASE 2A (PP2A) [41]. The transcriptional factor BES1/BZR1 affects plant growth and development in various aspects through the regulating expression of thousands of BR responsive genes. Among these genes, glycogen synthase kinase-3 (GSK3)-like kinase BIN2 is a key suppressor that regulates plant growth and development by determining the phosphorylation status of BES1 and BZR1 [33, 34, 39, 40]. GSK3-like kinases are a highly conserved Ser/Thr kinases that are implicated in a wide range of cellular and developmental processes [42]. In *Arabidopsis*, the GSK3/SHAGGY-like family has 10 gene members that can be classified into four subgroups [43]. In this family, the *Arabidopsis* GSK3-like kinase (AT4G18710, BIN2/UCU1/DWF12/AtSK21) which belongs to the group II, has activity to negatively regulate the BR signal transduction by phosphorylating BZR1/BES1 [39, 40, 44]. The gain-of-function *bin2* mutant was discovered to be insensitive to BRs in *Arabidopsis* and has the shaggy phenotypic characteristic of dwarfing architecture. It also confers curved leaves, and an impaired cell elongation [45]. The coding sequence of the *BIN2* gene, substitutes consecutive glutamate residues in the highly conserved TREE domain, which results in the negatively regulating growth by phosphorylating the BES1 and BZR1 proteins, that result in the degradation of BZR1 to reduce its activity [40]. Based on sequence similarity of BIN2 with its two closest group II *Arabidopsis* homologs, BIN2-Like1 (BIL1) and BIN2-Like2 (BIL2), which belong to the AtSKs group [40]. It was further suggested that BIL1 and BIL2 may also be involved in BR signaling. Overexpression of *BIL1* or *BIL2* gene driven by their native promoters in wild-type *Arabidopsis* plants exhibits the dwarf phenotype [46]. However, the evidence of *BIL1* and *BIL2* genes involved in BR signal transduction is still insufficient, and the mechanism of plant dwarf phenotype caused by overexpression of *BIL1* and *BIL2* genes remains to be elucidated. Therefore, it is urgent to further explore their participation and even related mechanism.

In this study, a pure dwarf mutant, *Bndwarf2*, was found in advanced selfing generation in a nearly pure line CB1501-1 in *B. napus*. To expedite this study, the dwarf gene *BnaC04.BIL1* was isolated using map-based cloning. The *BnaC04.BIL1* gene encoding a GSK3-like kinase, belongs to GSK II subfamily. Genetic transformation experiments confirmed that the *BnaC04.BIL1* was responsible for the plant dwarf phenotype in the *Bndwarf2* mutants. Our study clarifies the role of *BnaC04.BIL1* in the regulation of plant height, which may help to improve lodging resistance in oilseed rape.

## Results

### Characterization of the *Bndwarf2* Mutant

**Fig. 1:**
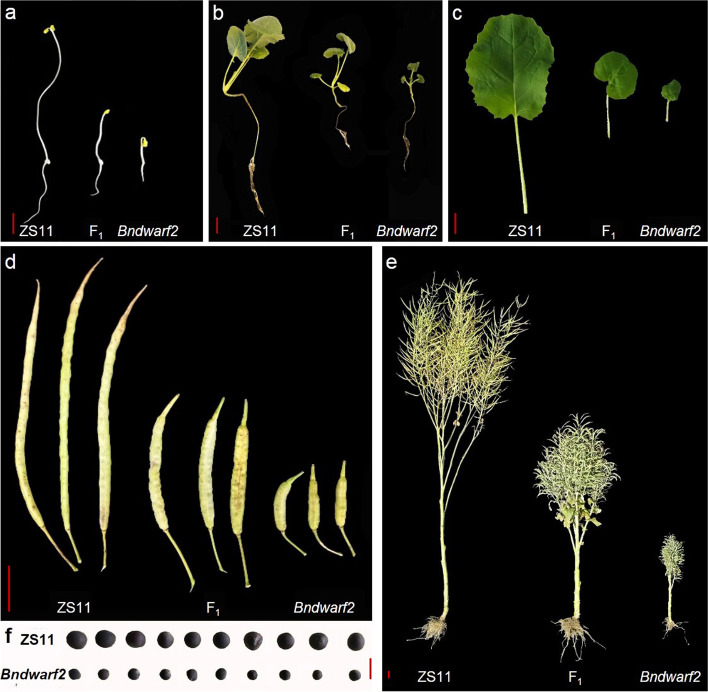
Phenotypic comparison among ZS11, F_1_, and *Bndwarf2* mutant. **a** The root length and hypocotyl length of ZS11 (left), F_1_ (middle), and *Bndwarf2* (right) in the dark for 6 d. **b** The performance of ZS11 (left), F_1_ (middle), and *Bndwarf2* (right) at seedling stage. **c** The petioles comparison at seedling stage. **d** The plant height comparison at maturity stage. **e** The siliques comparison of at maturity stage. **f** The seeds comparison of ZS11 (up) and *Bndwarf2* mutant (down). Bars = 2 cm

A pure dwarf mutant, *Bndwarf2* was obtained in advanced selfing generation in a nearly pure line CB1501-1 in *B. napus*. The *Bndwarf2* mutant showed obvious dwarf phenotype after 6 d dark germination compared to Zhongshuang 11 (ZS11, a conventional *B. napus* cultivar), which was used as a parent to map-based clone the gene responsible for the dwarfism (Fig. 1a). At seedling stage, the *Bndwarf2* mutant plants had shorter hypocotyls and shorter petioles (Fig. 1b, c). The leaves of *Bndwarf2* mutants showed darker green, thickened, and wrinkled leaves, and had significant higher Chl a, Chl b, and Chl contents than those of ZS11 (Table S1). At flowering stage, the *Bndwarf2* mutant showed significant difference in plant height from ZS11 (Figure S1). While at maturity stage, the *Bndwarf2* mutant showed dwarf stature (33.62 ± 1.12 cm) with no apical dominance, that was significantly lower than that for ZS11 (193.54 ± 4.80 cm) (Fig. 1d). The siliques of *Bndwarf2* mutants were significantly shorter compared to that of ZS11 (Fig. 1e). In addition, the *Bndwarf2* mutants had lower 1000-seeds weight and compact plant architecture (Fig. 1f; Table S2). The F_1_ plants (105.30 ± 5.16 cm) generated by cross of ZS11 with *Bndwarf2* were in-between that of ZS11 and *Bndwarf2*.

### Inheritance of the dwarf trait

To investigate the genetic regulation mechanism for *Bndwarf2*, the F_1_ (ZS11 × *Bndwarf2*) and RF_1_ (*Bndwarf2* × ZS11) plants were obtained by crossing *Bndwarf2* with ZS11, all had the dwarf trait, indicating that dwarf trait was controlled by dominant genes. The phenotypic segregation ratio of dwarf plants to tall plants in the F_2_ population was in a Mendelian model of 1:2:1 (69 homozygous dwarf plants vs. 140 hybrid dwarf plants vs. 78 tall plants, *χ*^*2*^ < *χ*^*2*^_0.05_) (Figure S2). Among 289 BC_1_ individuals, 139 as dwarf types and 150 as tall types, also approximately fitted an expected Mendelian inheritance ratio of 1:1 (dwarf plants vs. tall plants). In subsequent segregating F_2:3_ populations, the genetic regulation was confirmed (Table 1). These results indicated that the dwarf trait was controlled by a semi-dominant nuclear gene, which was named as *BnDWARF2* in the subsequent study.

**Table 1:** Inheritance of the plants height trait in populations derived from the two parents in *B. napus*

Population	Homozygous dwarf plants	Hybrid dwarf plants	Tall plants	Expectation	*χ*^*2*^	*P* value
F_1_	0	30	0			
RF_1_	0	30	0			
F_2_	69	140	78	1:2:1	0.74	0.69
BC_1_	0	139	150	1:1	0.35	0.56
F_2:3_	206	451	232	1:2:1	1.71	0.43

### Map-based cloning

**Fig. 2:**
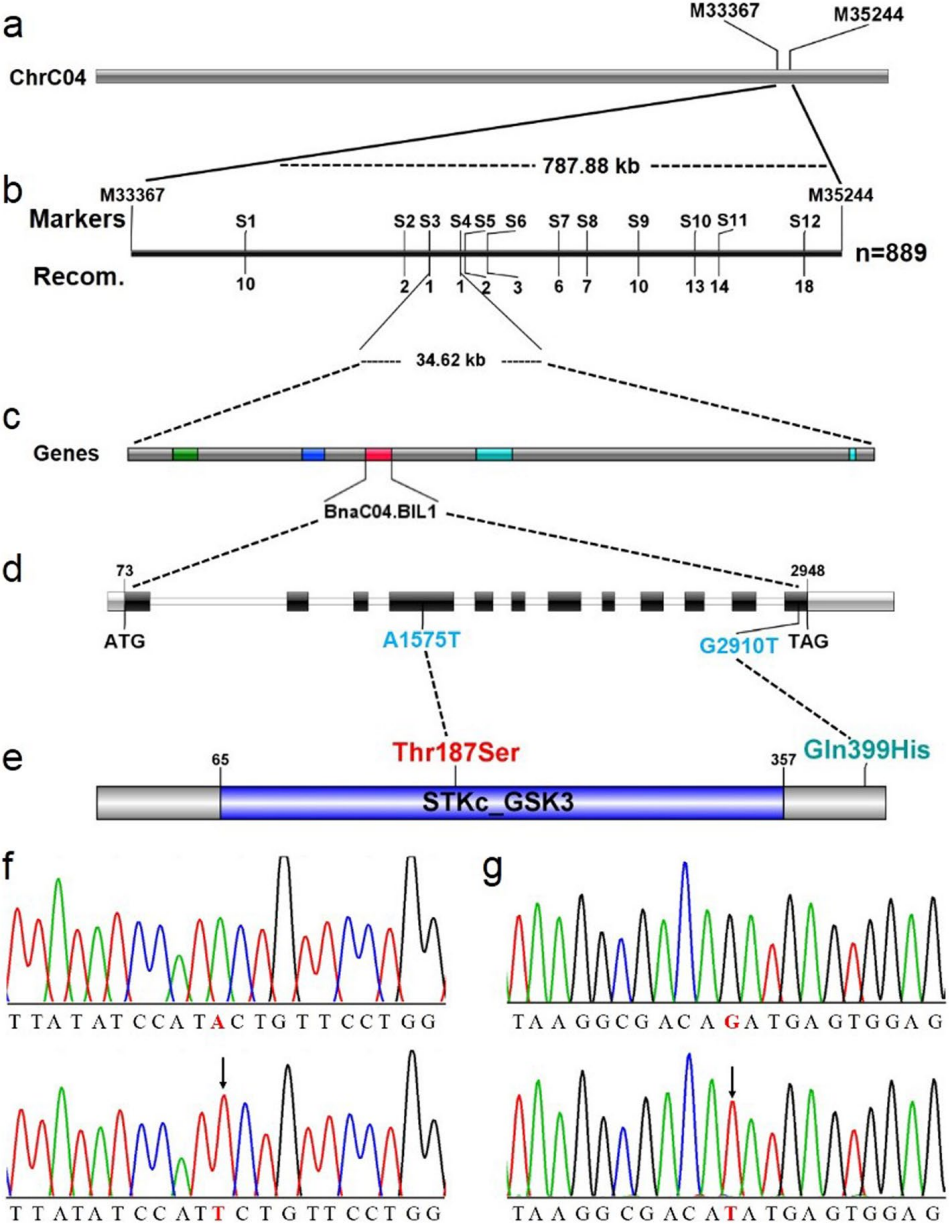
Map-based cloning of *BnDWARF2*. **a** The *BnDWARF*2 locus was mapped primarily on C04 chromosome between the SNP markers M33367 and M35244. **b** The *BnDWARF2* locus was fine-mapped in the 34.62 kb region between SSR markers S3 and S4. The numerals indicate the number of recombinants. **c** The genes in the mapping interval. **d** The gene structure and the mutation sites in *BnaC04.BIL1*. **e** The protein structure and the mutation sites of the BnaC04.BIL1 protein, and the STKc_GSK3 superfamily domain was predicted. Solid lines show the position of the amino acid transition. **f** The chromatogram of BnaC04.BIL1 at 1565–1585 bp in ZS11 and *Bndwarf2* mutant, respectively. **g** The chromatogram of BnaC04.BIL1 at 2900–2920 bp in ZS11 and *Bndwarf2* mutant, respectively. The black arrows denote the A1575T and G2910T substitutions, respectively

To map *BnDWARF2*, 94 plants (70 dwarf plants and 24 tall plants) from the F_2_ population were used for single nucleotide polymorphism (SNP) marker genotyping. Although the chip (Illumina, Inc) has 52,157 SNP markers, only 7457 polymorphic markers were used to construct the SNP genetic linkage map after removing the invalid or non-polymorphism markers. The *BnDWARF2* locus was primarily mapped within the 787.88-kb on C04 chromosome between the SNP marker M33367 and M35244 (Fig. 2a). To fine map the *BnDWARF2* locus, 318 primer pairs of simple sequence repeat (SSR) markers were designed to uniformly cover the preliminary mapping interval. A further 889 individuals from the F_2:3_ populations, finally narrowed down the *BnDWARF2* locus to a 34.62-kb region between SSR markers S3 and S4 (Fig. 2b). No other markers to further narrow the mapping interval were found for this mapping population and its parents. A total of 5 putative genes (*BnaC04g41640D*, *BnaC04g41650D*, *BnaC04g41660D*, *BnaC04g41670D*, and *BnaC04g41680D*) were localized in the 34.62-kb region according to the gene annotation of the *B. napus* reference genome (Fig. 2c; Table 2). Gene cloning was performed for the mapping interval, and the results showed that only *BnaC04g41660D* (*BnaC04.BIL1*) gene had 10 SNPs differences between ZS11 and *Bndwarf*2. The BnaC04.BIL1 had two amino acid residues substitutions at aa-187 (Thr-to-Ser mutation, named Thr187Ser) and aa-399 (Gln-to-His mutation, named Gln399His) (Fig. 2e).

**Table 2:** Information of 5 putative genes in the mapping interval

Gene in *B. napus*	Homologue in *A. thaliana*	Gene function
*BnaC04g41640D*	AT2G29770.1	Galactose oxidase/kelch repeat superfamily protein
*BnaC04g41650D*		unknown protein
*BnaC04g41660D*	AT2G30980	Encodes a GSK3-like protein kinase
*BnaC04g41670D*	AT2G30990.1	Arginine N-methyltransferase, putative (DUF688)
*BnaC04g41680D*		unknown protein

*BnaC04.BIL1* contains a 1233-bp open reading frame (ORF) with 11 introns in *B. napus* (Fig. 2d; Figure S3). *BnaC04.BIL1* is a homologous gene of the *Arabidopsis* AT2G30980 gene, which encodes a GSK3-like [47]. The conservative domain analysis showed that the amino acid sequence 65–357 was the conserved domain of STKc_GSK3, and Thr187Ser is in the conserved domain (Fig. 2e). The amino acid multiple sequence analysis showed that BnaC04.BIL1 had a series of amino acid residues conserved in GSK3 kinase, such as GSK3 domain signature SYICSR and plant-specific TREE motif (Figure S3a). It was perfectly aligned with the genes for GSK3/Shaggy kinases with regarding to a series of amino acid residues such as the GSK3 signature SYICSR within domain VIII that was absent from MAP kinase sequences [48]. The E-K mutation in the highly conserved TREE motif is thought to preventing the BR-mediated BIN2 inhibition [49], thus resulting in the increased BIN2 stability [50, 51]. The phylogenetic tree clustering and construction were analyzed by MEGA 7.0 selection Neighbor-joining method. The results showed that *BnaC04.BIL1* and *Arabidopsis BIN2* were homologous, belonging to GSK3 II subfamily (Figure S3b). These suggested that the *BnaC04.BIL1* gene may be responsible for the dwarf trait of *Bndwarf2*.

### Expression patterns of *BnaC04.BIL1* and the subcellular localization

**Fig. 3:**
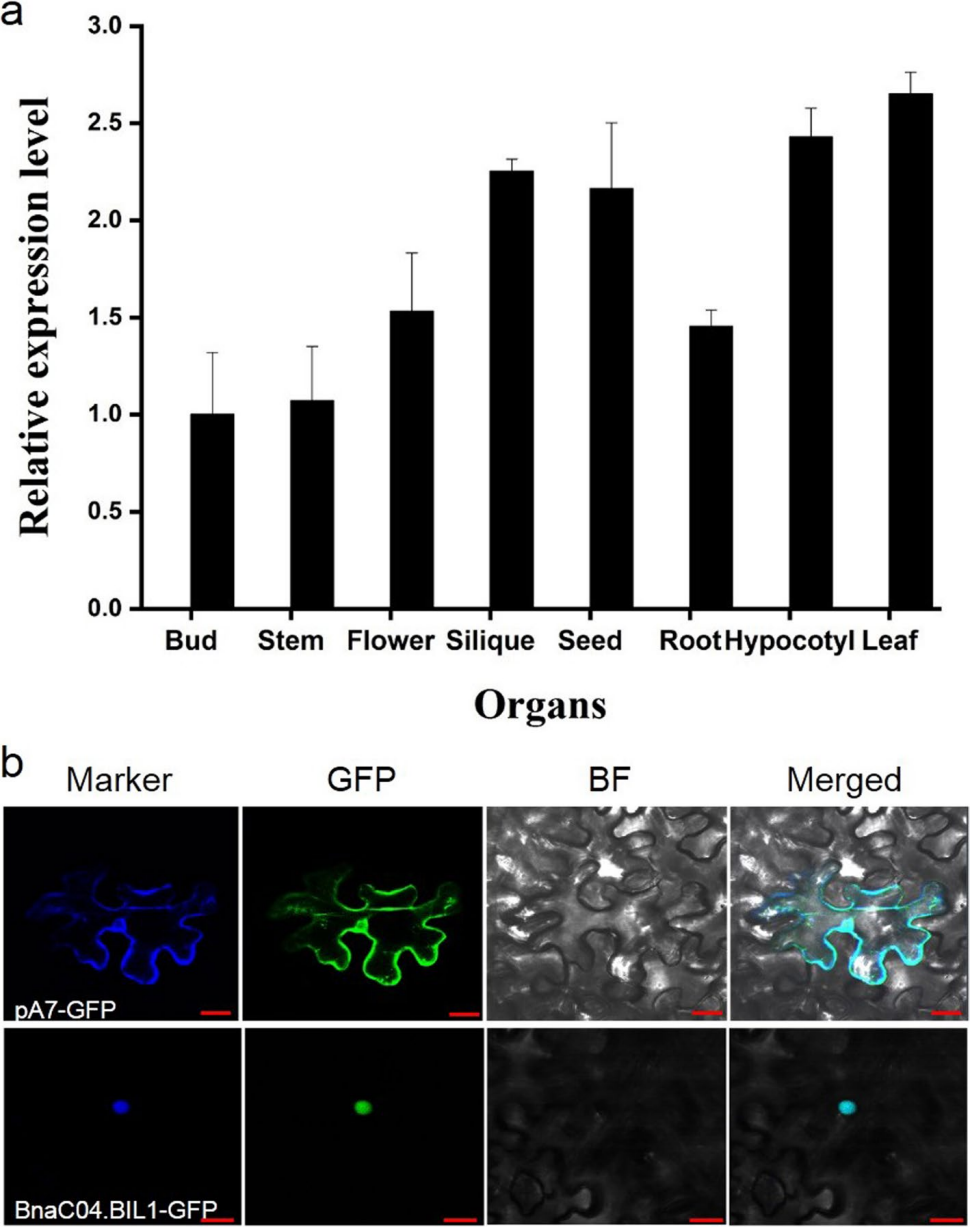
Expression pattern of *BnaC04.BIL1* and subcellular localization of its encoding protein. **a** Expression pattern of *BnaC04.BIL1* detected by qRT-PCR in bud, stem, flower, silique, seed, root, hypocotyl, and leaf from *Bndwarf2*. The *BnActin* gene was used as a reference gene and the expression level of bud was set to 1. The bud, stem, and flower samples are from flowering stage. The silique samples are from podding stage. The seed samples are from maturity stage. The root and hypocotyl samples are from 7-day-old seedlings grown on medium, and the leaf samples are from seedling stage. **b** Subcellular localization of BnaC04.BIL1 protein in tobacco leaf cells. Plasmids pA7-GFP and BnaC04.BIL1-GFP were introduced into tobacco leaf cells by particle bombardment, respectively. Bars = 20 μm

To explore the possible function of *BnaC04.BIL1* gene from *Bndwarf2* mutant in different tissues, the transcription levels of *BnaC04.BIL1* in leaves, roots, hypocotyls, stems, buds, flowers, siliques, and seeds were analyzed. The qRT-PCR analysis showed that the *BnaC04.BIL1* gene was expressed in all tissues, which indicated that *BnaC04.BIL1* expressed constitutively (Fig. 3a). The expression level of *BnaC04.BIL1* was higher in leaves, hypocotyls, siliques, and seeds, while its level in buds and stems were lower.

Previous research showed that *Arabidopsis* BIN2 [52] was localized in the nucleus. To define the subcellular location of expression, pA7-GFP and BnaC04.BIL1-GFP constructs were then introduced into the tobacco leaf cells by the particle bombardment method. The merged image of BnaC04.BIL1-GFP and nuclear localization signal (NLS)-mCherry signals showed that BnaC04.BIL1 was localized to the nucleus (Fig. 3b). The result showed the *BnaC04.BIL1* gene functions in the nucleus.

### Overexpression of *BnaC04.BIL1* leads to plant dwarf

**Fig. 4:**
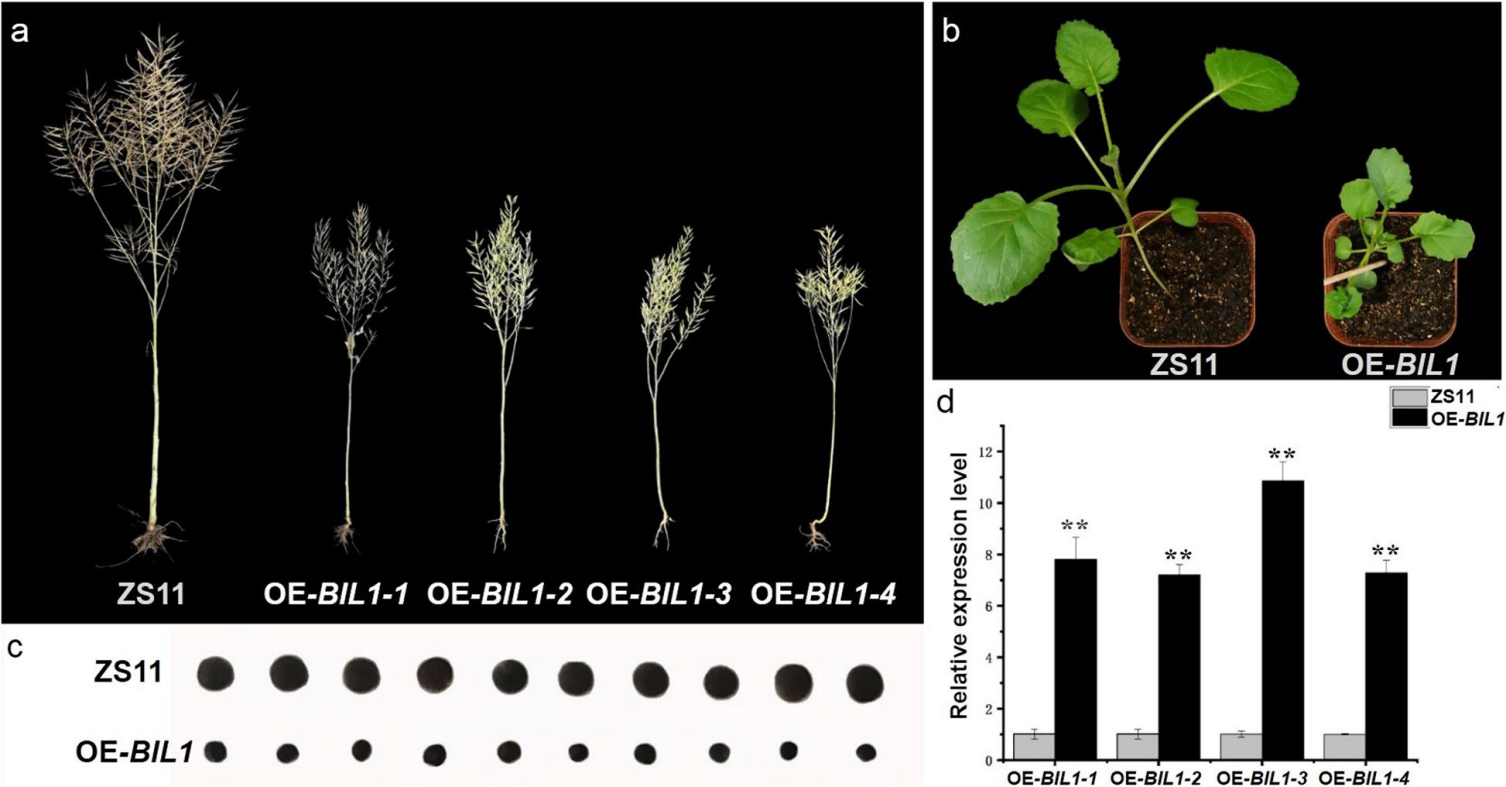
Phenotype comparison and qRT-PCR analysis between the ZS11 and OE*-BIL1* transgenic plants. **a** The phenotype of ZS11 (left) and OE*-BIL1* (right) transgenic plants at maturity stage. **b** The phenotype of ZS11 (left) and OE*-BIL1* (right) transgenic plants at seedling stage. **c** The seeds of ZS11 (up) and OE*-BIL1* (down) plants. **d** qRT-PCR analysis of *BnaC04.BIL* in ZS11 and OE*-BIL1* transgenic lines

To investigate *BnaC04.BIL1* functioning in plant height, a construct was generated by inserting a 1233 bp *BnaC04.BIL1* ORF fragment from *Bndwarf2* into the vector pBI-121 under the control of the CaMV35S promotor. The construct was introduced into ZS11 plants by *Agrobacterium*-mediated transformation. The plant height trait was compared between the ZS11 and OE*-BnaC04.BIL1* (OE*-BIL1*) transgenic plants by overexpressing the *BnaC04.BIL1* gene. Notably, plant height in the OE*-BIL1* transgenic plants was similar to the expected *Bndwarf2* phenotype with obvious dwarf stature; meanwhile, the transgenic plants also displayed dramatically smaller seeds than the ZS11 plants (Fig. 4a-c; Table S3). At the seedling stage, the OE*-BIL1* transgenic lines displayed darker green and wrinkled leaves compared to those of ZS11 (Fig. 4b). These results suggest that the *BnaC04.BIL1* gene not only controls the plant height, but also regulates the seed size. It follows that, the yield of per OE*-BIL1* transgenic plants showed a significantly reduction compared to that of ZS11 (Table S3). The T_2_ progeny plants were examined from six independent T_1_ transgenic lines in growth chamber, which showed the expected Mendelian inheritance ratio of 3:1 in T_2_ progeny (dwarf vs. tall plants, *χ*^*2*^ < *χ*^*2*^_0.05_, 1 = 3.84; *P* > 0.05; Table S4). The T_2_ progeny plants displayed perfect co-segregation between the transgene and the dwarf phenotype. Consistently, the expressions of *BnaC04.BIL1* gene in homozygous T_3_ lines (OE*-BnaC04.BIL1* transgenic genes) were significantly higher than those of ZS11 plants (Fig. 4d). These results confirmed that the *BnaC04.BIL1* is the causal mutation for the dwarfism and controls smaller seeds, which were also observed in *Bndwarf2*.

## Discussion

Plant height is an important growth habit that is a fundamental yield determining trait in crops. In the 1960s and 1970s, the dwarf trait genes (*Rht1* and *sd1*) were introduced into wheat and rice that were crucial to the first “Green Revolution” [1, 53]. The semi-dwarf architecture can help to increase harvest index, increase planting density, enhance lodging resistance, and thus be suitable for mechanization harvest [54]. However, there are few studies with respect to dwarf oilseed rape. Because of the lower mechanization level of oilseed rape production and few varieties suitable for mechanization harvest, oilseed rape production faces severe challenge.

Most of our knowledge about BIN2 functions came mostly from gain-of-function results. For example, genetic screening in *Arabidopsis* for BR-insensitive dwarf mutants resulted in the isolation of eight gain-of-function *bin2* alleles [39, 40, 45]. Based on sequence similarity of BIN2 with its two closest group II *Arabidopsis* homologs, BIN2-Like1 (BIL1) and BIN2-Like2 (BIL2), which belong to the AtSKs group [46]. Overexpression of *BIL1* or *BIL2* gene driven by their native promoters in wild-type *Arabidopsis* plants exhibits the dwarf phenotype [46]. In our study, a gain-of-function mutation for *BIL1* in oilseed rape has been discovered, and most importantly, it exhibits the BR-insensitive dwarf phenotype. For example, the *Bndwarf2* mutant displayed the BR signaling phenotypes: shorter hypocotyls, shorter petioles, wrinkled leaves, and obvious dwarf compared with the ZS11 (Fig. 1; Figure S1; Table S2). These characteristics were similar to the phenotypes of BR-insensitive mutants such as *bri1* [27], *dwf12* [39], and *ucu1* [45]. Through map-based cloning, the BnaC04.BIL1 was identified to be a *BIN2-Like1* (*BIL1*), showing a Thr187Ser amino acid substitution residing in the conserved region (Fig. 2; Figure S3a). Genetic transformation experiments confirmed that the *BnaC04.BIL1* was responsible for the plant dwarf phenotype in the *Bndwarf2* mutants. Overexpression of *BnaC04.BIL1* under the background of ZS11 reduced plant height compared with ZS11 (Fig. 4; Table S3). This result was consistent with previous reports, showing that overexpressing *BIL1* gene confers the dwarf phenotype in *Arabidopsis* [46]. The genetic evidence clarifies the *BnaC04.BIL1* can sharply change plant architecture in natural plant accessions in allotetraploid.

Further study has identified *Bndwarf2*, a dwarf and compact mutant in *B. napus*, and the dwarf trait is controlled by a semi-dominant nuclear gene (Table 1). The plant height of F_1_ derived from the cross of *Bndwarf2* with the tall parent, decreased by about 50% compared to that of tall plant (Table S2). Particularly, the *Bndwarf2* displayed an extreme reduction in height at maturity, which is different from the previously reported dwarf mutants in *B. napus* [4, 8, 55–57]. For example, the dwarf locus of *bnC.dwf* mutant was controlled by a recessive gene [56]. And, the dwarf trait of *Bndwf1* mutant was controlled by a semi-dominant gene [8]. The F_1_ plants have compact properties such as shortened branch, shortened gap between siliques, shortened gap between branches and dwarfing plant height by *BnDWARF2* gene (Fig. 1; Figure S1; Table S2). This finding implicates that the plant architecture of homozygous or heterozygous individuals derived *Bndwarf2* mutant is compact (Fig. 1). This kind of compact architecture can be undoubtedly helpful to increase planting density, enhance lodging resistance and increase planting density, therefore the compact plant architecture is ideal for machinery production of oilseed rape.

The germplasm *Bndwarf2* has compact plant type, and lacks strong growth vigor. However, the compact plant architecture can be used in hybrid cultivar development in which the compact type and hybrid vigor can be combined well. This is helpful to breeding of variety breeding with the objectives such as high-yield, good quality and suitable for machinery. On the other hand, the growth vigor in pure line or cultivar may be improved in some genetic background. Some reports have demonstrated that the genes in BIN2 regulation network can also interact with BIN2, leading to improvement of the growth inhibition caused by BIN2 gene overexpression caused by natural biological accession state or by transgenic [58–61]. We speculate that some gene may interact with BnaC04.BIL1 to attenuate its role in limit growth vigor as that the *Arabidopsis* homolog BIN2 crosstalk experiments have shown. Furthermore, expressions of some regulator genes may probably alter the expression level of *BnaC04.BIL1* that is constitutively expressed in the various organs, and reduced expression level may improve the growth vigor. The subcellular localization analysis demonstrated that BnaC04.BIL1 exists in the nucleus (Fig. 3). Consistently, the *Arabidopsis* BIN2 functioned in nucleus to negatively regulate BR signaling [52]. In fact, previous results revealed that many genes regulated by BZR1 and/or BES1, and some proteins interacting with BZR1/BES1, were closely associated with the BR signaling [29, 62]. The BR signal transduction pathways was impairment to lead to the dwarfing phenotype.

## Methods

### Plant materials and growth conditions

A pure dwarf mutant, *Bndwarf2* was found in advanced selfing generation in a nearly pure line CB1501-1 in *B. napus* from our germplasm bank of our lab in Nanjing Agricultural University. The populations for mapping the *BnDWARF2* locus*,* were generated from the crosses between *Bndwarf2* and the canola variety Zhongshuang 11 (ZS11). All oilseed rape materials were grown in growth chamber and the fields of the Jiangpu Agricultural Experimental Station at Nanjing Agricultural University.

Tobacco was grown in growth chamber. The illumination period was 14 h with temperature at 26 °C and 10 h with temperature at 20 °C. When tobacco leaves at 5-leaf stage were used for the subcellular localization.

### Map-based cloning

SNP and SSR markers were used to map the dwarf gene. 70 dwarf plants, 24 tall plants and parents from F_2_ population were genotyped using a Brassica 60 K SNP Bead Chip Array (Illumina, Inc), which have a total of 52,157 SNP markers. The SNP genetic map was constructed by JoinMap 4.1 mapping software [63], then the *BnDWARF2* locus was primarily mapped onto physical and genetic map. The mapping interval sequence was downloaded from the *Brassica napus* Genome Browser (http://www.genoscope.cns.fr/brassicanapus/cgi-bin/gbrowse/colza/). Using this genomic sequence, SSR marker primers were designed by aid of SSR Hunter 1.3 [64], and Primer Premier 5.0 [65]. A total of 318 polymorphic SSR markers were obtained. These SSR markers helped to fine-map the *BnDWARF2* locus using a size-enlarged population comprised of F_2:3_ plants.

To identify genes associated to the dwarf trait, sequence of the fine mapping interval was obtained from the *Brassica napus* Genome Browser for reference to next-step experiments. Then, all of the genes in the fine mapping interval were cloned from *Bndwarf2* and parent ZS11. And, the resulting sequences were aligned using ClustalX 1.83 and GeneDoc software. The specific primers of the genes are listed in Table S5.

### Sequence analysis

The *B. napus* BIL1 genes were obtained by screening the *B. napus* Genome Browser (http://www.genoscope.cns.fr/brassicanapus/) with known *A. thaliana* BIL1 gene as a query. The Conserved Domain Database was used to search the protein functional in the National Center for Biotechnology Information (NCBI) (http://www.ncbi.nlm.nih.gov). Predicted *A. thaliana* BIL1 amino acid sequences were obtained from the TAIR website (http://www.arabidopsis.org/Blast). Moreover, the protein sequences of other species were obtained from the NCBI using the *A. thaliana* BIL1 protein sequence as a query. All obtained protein sequences were aligned using ClustalX 1.83 [66]. Additionally, a phylogenetic tree was constructed using MEGA 7.0 [67] with maximum likelihood method, and the bootstrap values were estimated with 1000 replicates.

### RNA extraction and qRT-PCR

Total RNA was extracted from various samples using TRIzol reagent (Sigma; http://www.sigmaaldrich.com/). First-strand cDNA synthesis was carried out using a Reverse Transcription System (Takara, Tokyo, Japan). The cDNA was used as the template for qRT-PCR analysis with specific primers (Table S5). The qRT-PCRs were carried out with SYBR Green Real-time PCR Master mix using a CFX96-2 PCR machine (BIO-RAD, USA). Relative expression levels were calculated using the 2^−ΔΔCt^ method with Actin as an internal control.

### Plant transformation

The 1223-bp *BnaC04.BIL1* open reading frame was amplified from *Bndwarf2* using the primers *BnaC04.BIL1*-F/R (Table S5) and cloned into the *Xba* I-*Bam*H I sites of the overexpression pBI121 vector with CaMV35S promotor to construct the 35S::*BnaC04.BIL1*-pBI121 plasmid. The 35S::*BnaC04.BIL1*-pBI121 plasmid was introduced into *Agrobacterium tumefaciens* strain EHA105 by a heat shock method. The positive *A. tumefaciens* were transformed into ZS11 with a modified floral dip method. Briefly, agrobacteria cultures carrying a target construct were collected by centrifugation and then resuspended in a solution containing 1/2 MS salts containing 3% Suc, 0.1% Silwet L-77, 2 ng/L 6-benzyladenine, and 8 mg/L acetosyringone. The ZS11 plants at the flowering stage were used for the transformation. The head of a flowering plant was bent downward and dipped into a beaker containing the agrobacterial culture liquid for 3 min, and the treated plant head was loosely wrapped with a vegetable parchment paper. The plant for transformation was treated every week 1 to 2 times and then continued to grow until maturation. Seeds that experienced transgenic treatment were harvested. The transformant leaf were collected for PCR detection [68]. The 35S::*BnaC04.BIL1*-pBI121 sequence was detected by PCR in transgenic plants, the transgenic plants were named OE-*BnaC04.BIL1* (*OE-BIL1*).

## Supplementary Information

**Additional file 1: Figure S1. **The plant height of ZS11, F_1_, and* Bndwarf2* at flowering stage. **Figure S2.** The distribution of plant height in the F_2_ population. **Figure S3.** Sequence analysis of BnaC04.BIL1 protein. **a** Multiple sequence alignment of amino acid sequences of BnC04.BIL1 protein. The conserved TREE and SYICSR motif were boxed in red. At, Cs, Cr, Aa, Es, Bo, Br, Bn, Cs, Cc, Qs, Pp, Hu, Cp, Ap, Gm, Vv, Ac, and Mn denote *Arabidopsis thaliana*, *Camelina sativa*, *Capsella rubella*, *Arabis alpine*, *Raphanus sativus*, *Eutrema salsugineum*, *Brassica oleracea*, *Brassica rapa*, *Brassica napus*,*Citrus sinensis*, *Quercus suber*, *Prunus persica*,*Herrania umbratica*, *Carica papaya*, *Abrus precatorius*, *Glycine max*, *Vitis vinifera*, *Actinidia chinensis*, *Morus notabilis*, respectively.** b** Phylogenetic tree analysis of BnaC04.BIL1 using Neighbor-joining method in MEGA 7.0 program. Bootstrap values from 1000 replicates were indicated at each node. The GSK3 group I is marked with green, and the GSK3 group II is marked with red, and the GSK3 group III is marked with yellow, and the GSK3 group IV is marked with blue. The BnaC04.BIL1, BnaC04.BIL1-Mut, and BnaC04.BIL1-WT are labeled. **Table S1. **Leaf chlorophyll contents in the leaves of ZS11 and* Bndwarf2* mutant. **Table S2.** Agronomic trait comparisons in ZS11, F_1_, and* Bndwarf2*. **Table S3.** Agronomic traits of OE*-BIL1* transgenic lines. **Table S4. **Genetic analysis of T_2_ progeny derived from six independent T_1_transgenic plants.

**Additional file 2: Table S5.** Primers used in this study.

## Data Availability

The datasets generated and/or analyzed during the current study are available in the Figshare repository (https://doi.org/10.6084/m9.figshare.14679021).

## Abbreviations

*B. napus*: *Brassica napus*
GSK3: GLYCOGEN SYNTHASE KINASE 3
BIN2: BRASSINOSTEROID INSENSITIVE2
BIL1: BIN2-LIKE1
qRT-PCR: quantitative real-time PCR
ZS11: Zhongshuang 11
SNP: Single nucleotide polymorphism
SSR: Simple sequence repeat
Thr187Ser: Thr-to-Ser mutation at 187
Gln399His: Gln-to-His mutation at 399
GA: gibberellin
BR: brassinosteroid
CYP: cytochrome P450 monooxygenase
CPD: CONSTITUTIVE PHOTOMORPHOGENESIS AND DWARFISM
BRD1: BR*-*DEFICIENT DWARF 1
DET2: DE-ETIOLATED 2
BRI1-BAK1: BR-INSENSITIVE1/BRI1-ASSOCIATED KINASE 1
BZR1/BES1: BRASSINAZOLERESISTANT1/BRI1-EMS-SUPPRESSOR1
CDG1: CONSTITUTIVE -DIFFERENTIAL GROWTH 1
BSK1: BR SIGNALING KINASE1
BSU1: BRI-SUPPRESSOR1
PP2A: PROTEIN PHOSPHATASE 2A
ORF: Open reading frame
OE*-BIL1*: OE*-BnaC04.BIL1*
NCBI: National Center for Biotechnology Information
