# Supplementary material for: Fine mapping of the BnaC04.BIL1 gene controlling plant height in Brassica napus L
Source: BMC Plant Biol. 2021 Aug 5;21:359. doi: 10.1186/s12870-021-03137-9 (PMC8340546; doi:10.1186/s12870-021-03137-9)
Supplement: Supplementary file 1 — Additional file 1: Figure S1. The plant height of ZS11, F1, and Bndwarf2 at flowering stage. Figure S2. The distribution of plant height in the F2 population. Figure S3. Sequence analysis of BnaC04.BIL1 protein. a Multiple sequence alignment of amino acid sequences of BnC04.BIL1 protein. The conserved TREE and SYICSR motif were boxed in red. At, Cs, Cr, Aa, Es, Bo, Br, Bn, Cs, Cc, Qs, Pp, Hu, Cp, Ap, Gm, Vv, Ac, and Mn denote Arabidopsis thaliana, Camelina sativa, Capsella rubella, Arabis alpine, Raphanus sativus, Eutrema salsugineum, Brassica oleracea, Brassica rapa, Brassica napus,Citrus sinensis, Quercus suber, Prunus persica,Herrania umbratica, Carica papaya, Abrus precatorius, Glycine max, Vitis vinifera, Actinidia chinensis, Morus notabilis, respectively. b Phylogenetic tree analysis of BnaC04.BIL1 using Neighbor-joining method in MEGA 7.0 program. Bootstrap values from 1000 replicates were indicated at each node. The GSK3 group I is marked with green, and the GSK3 group II is marked with red, and the GSK3 group III is marked with yellow, and the GSK3 group IV is marked with blue. The BnaC04.BIL1, BnaC04.BIL1-Mut, and BnaC04.BIL1-WT are labeled. Table S1. Leaf chlorophyll contents in the leaves of ZS11 and Bndwarf2 mutant. Table S2. Agronomic trait comparisons in ZS11, F1, and Bndwarf2. Table S3. Agronomic traits of OE-BIL1 transgenic lines. Table S4. Genetic analysis of T2 progeny derived from six independent T1transgenic plants. [file 12870_2021_3137_MOESM1_ESM.docx]

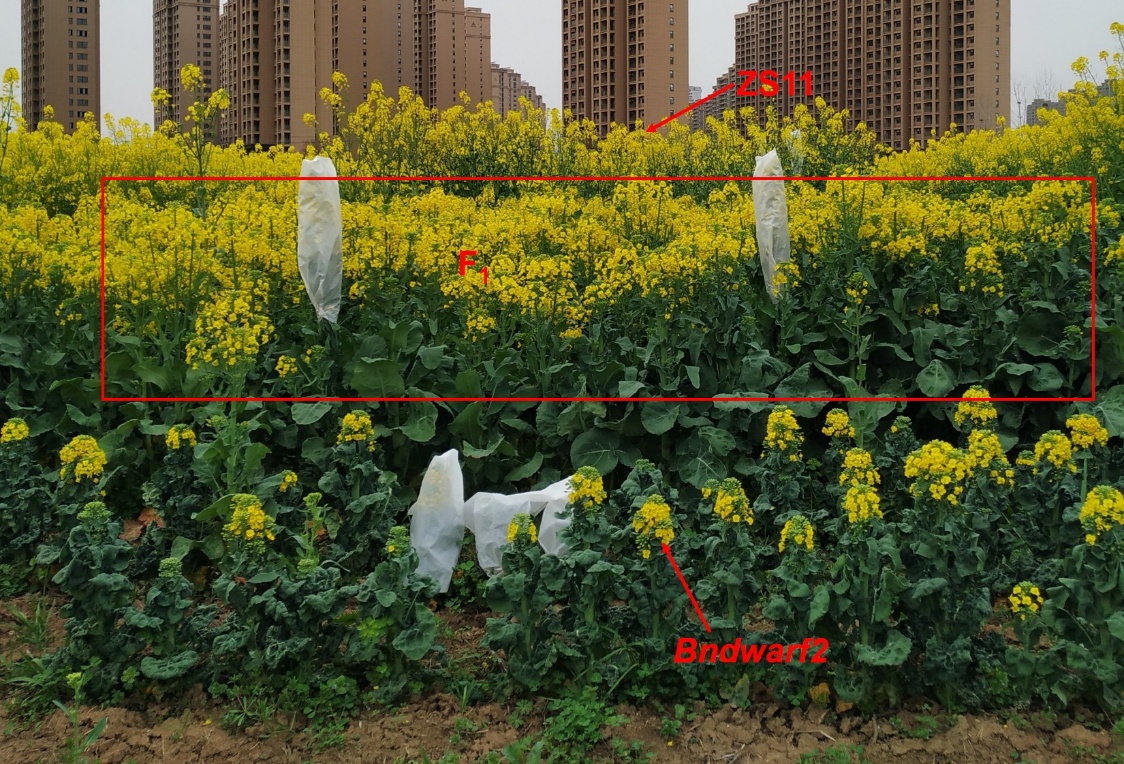


**Figure S1** The plant height of ZS11, F_1_, and *Bndwarf2* at flowering stage


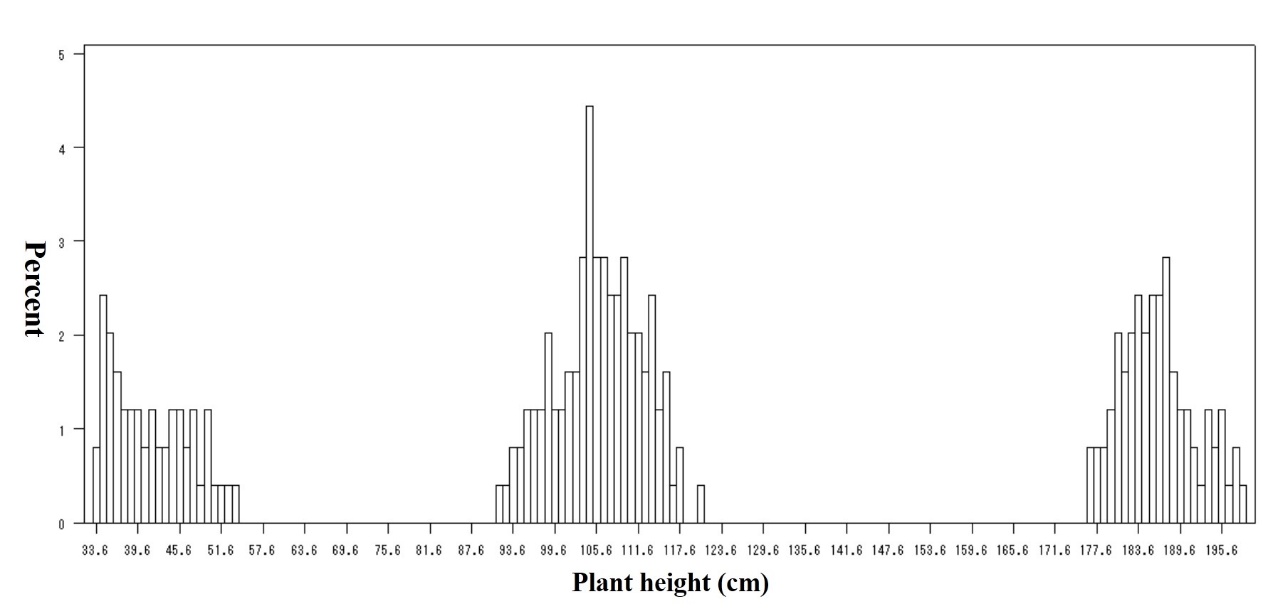


**Figure S2** The distribution of plant height in the F_2_ population


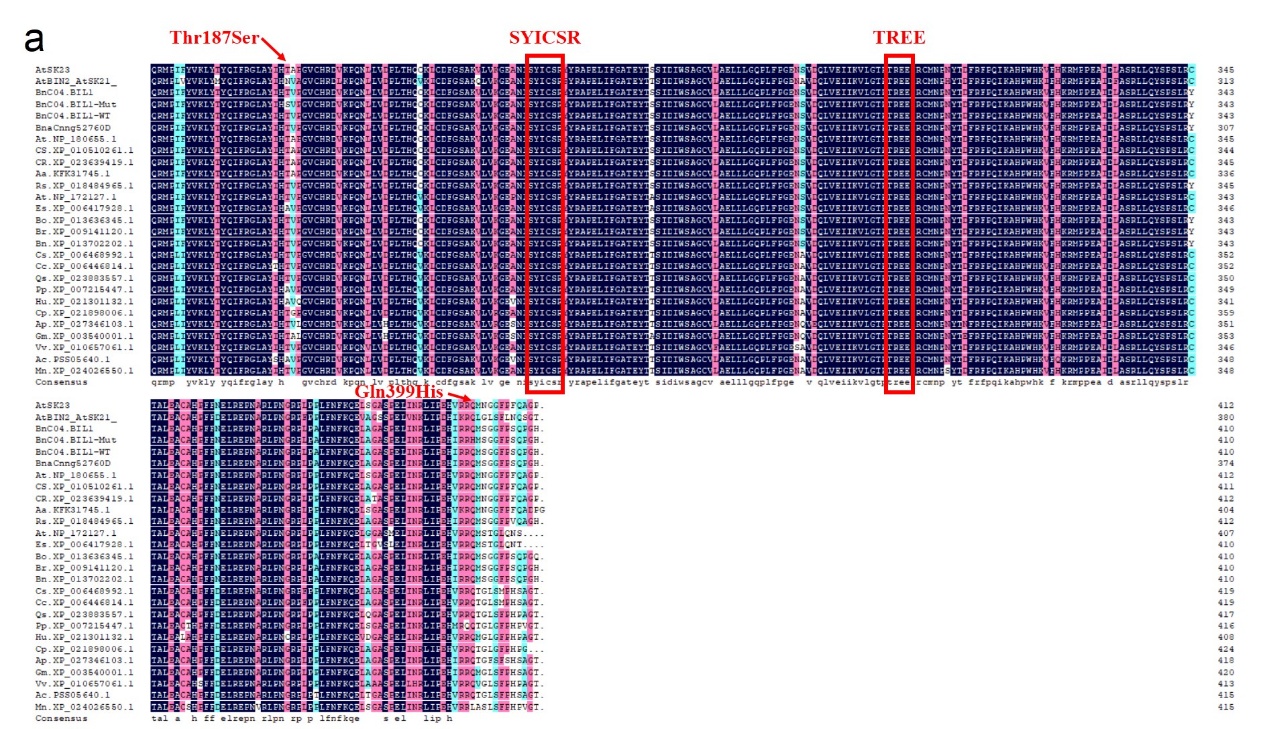


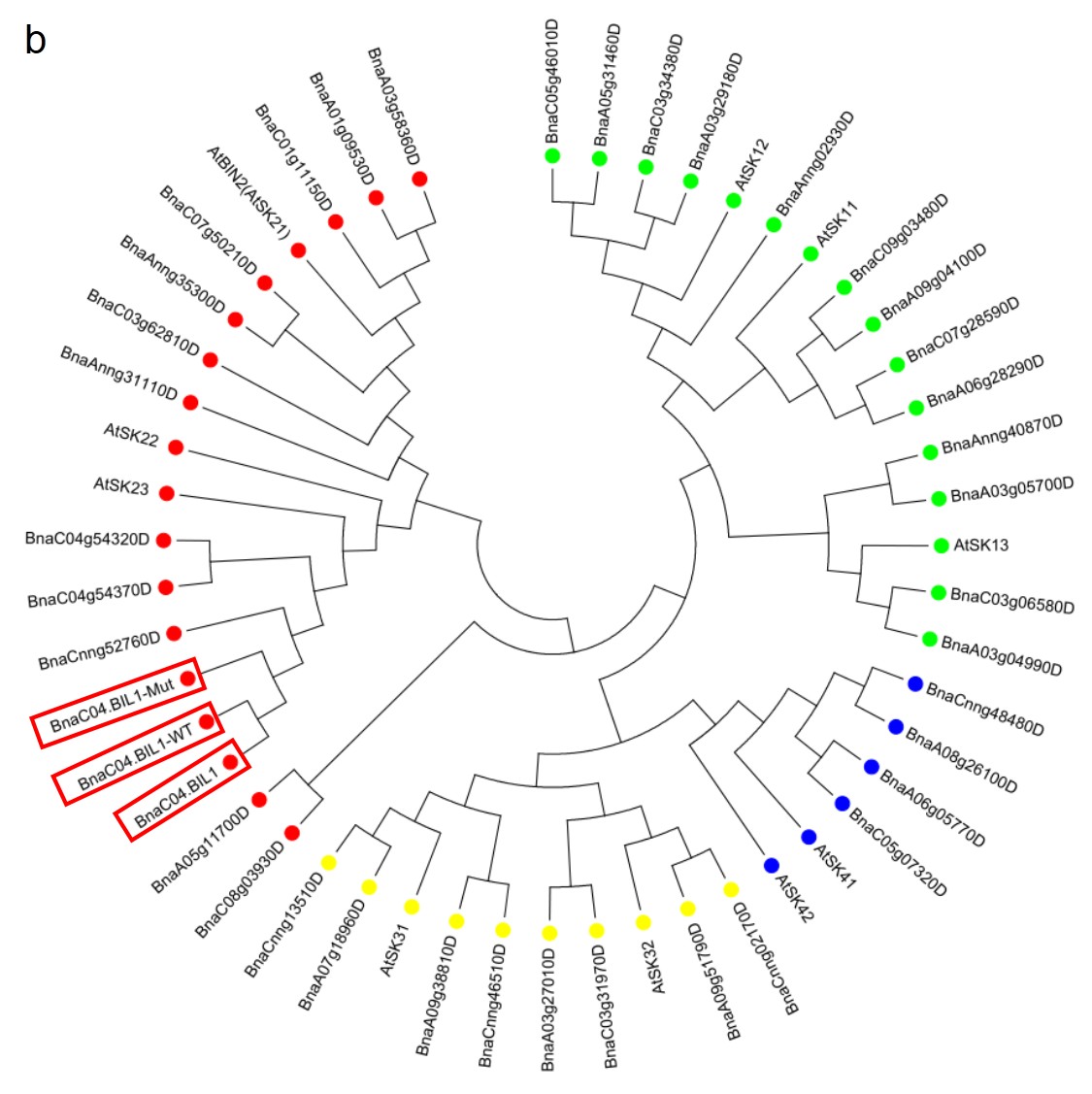


**Figure S3** Sequence analysis of BnaC04.BIL1 protein. **a** Multiple sequence alignment of amino acid sequences of BnC04.BIL1 protein. The conserved TREE and SYICSR motif were boxed in red. At, Cs, Cr, Aa, Es, Bo, Br, Bn, Cs, Cc, Qs, Pp, Hu, Cp, Ap, Gm, Vv, Ac, and Mn denote *Arabidopsis thaliana*, *Camelina sativa*, *Capsella rubella*, *Arabis alpine*, *Raphanus sativus*, *Eutrema salsugineum*, *Brassica oleracea*, *Brassica rapa*, *Brassica napus*, *Citrus sinensis*, *Quercus suber*, *Prunus persica*, *Herrania umbratica*, *Carica papaya*, *Abrus precatorius*, *Glycine max*, *Vitis vinifera*, *Actinidia chinensis*, *Morus notabilis*, respectively. **b** Phylogenetic tree analysis of BnaC04.BIL1 using Neighbor-joining method in MEGA 7.0 program. Bootstrap values from 1000 replicates were indicated at each node. The GSK3 group I is marked with green, and the GSK3 group II is marked with red, and the GSK3 group III is marked with yellow, and the GSK3 group IV is marked with blue. The BnaC04.BIL1, BnaC04.BIL1-Mut, and BnaC04.BIL1-WT are labeled

**Table S1** Leaf chlorophyll contents in the leaves of ZS11 and *Bndwarf2* mutant

| Genotype | Chl a (mg/g) | Chl b (mg/g) | Total Chl (mg/g) | Chl a/b ratio |
| --- | --- | --- | --- | --- |
| ZS11 | 1.32±0.20 | 0.75±0.10 | 2.07±0.29 | 1.77±0.13 |
| *Bndwarf2* | 2.24±0.19* | 1.24±0.17* | 3.48±0.32* | 1.83±0.23 |

^*^ indicates significant at the 0.05 probability level. Mean ± standard deviation (*SD*) (*n*=16 for each sample).

**Table S2** Agronomic trait comparisons in ZS11, F_1_, and *Bndwarf2*

| Trait | ZS11 | F_1_ | *Bndwarf2* |
| --- | --- | --- | --- |
| Plant height (cm) | 193.54±4.80 | 105.30±5.16 | 33.62±1.12 |
| Branching height (cm) | 45.28±5.65 | 40.83±3.76 | 23.16±1.89 |
| Length of main inflorescence (cm) | 74.32±6.66 | 40.68±5.54 | 8.89±1.26 |
| Stem diameter (mm) | 24.26±0.43 | 23.54±0.23 | 16.22±0.37 |
| Number of first effective branch | 7.56±1.18 | 5.70±0.84 | 4.36±1.92 |
| Siliques of main inflorescence | 87.60±6.27 | 75.00±5.43 | 33.60±8.29 |
| Total siliques per plant | 541.40±66.46 | 358.40±54.43 | 132±22.18 |
| Silique length | 10.33±0.12 | 5.71±0.15 | 2.36±0.08 |
| Seeds per silique | 29.06±2.20 | 24.50±2.38 | 9.26±2.24 |
| 1000-seed weight (g) | 4.23±0.14 | 3.08±0.35 | 2.46±0.23 |
| Yield per plant (g) | 66.55±7.72 | 27.02±6.32 | 3.09±5.58 |

ZS11 indicates tall plants. Data are shown as mean ± standard deviation (*n*=30 for each sample).

**Table S3** Agronomic traits of OE*-BIL1* transgenic lines

| Trait | OE*-BIL1* |
| --- | --- |
| Plant height (cm) | 112.30±5.80 |
| Branching height (cm) | 45.40±7.07 |
| Length of main inflorescence (cm) | 48.48±4.27 |
| Stem diameter (mm) | 11.98±1.30 |
| Number of first effective branch | 5.60±0.89 |
| Siliques of main inflorescence | 71.00±8.43 |
| Total siliques per plant | 188.40±62.49 |
| Silique length | 4.70±0.92 |
| Seeds per siliques | 10.89±2.08 |
| 1000-seed weight (g) | 2.13±0.16 |
| Yield per plant (g) | 5.33±1.38 |

Data are shown as mean ± standard deviation (*n*=15 for each sample).

**Table S4** Genetic analysis of T_2_ progeny derived from six independent T_1_ transgenic plants

| Population | OE1 | OE2 | OE3 | OE4 | OE5 | OE6 |
| --- | --- | --- | --- | --- | --- | --- |
| Number of dwarf plants | 41 | 35 | 40 | 39 | 42 | 43 |
| Number of tall plants | 17 | 16 | 13 | 17 | 12 | 14 |
| Total plants | 58 | 51 | 53 | 56 | 54 | 57 |
| Expected ratio | 3:1 | 3:1 | 3:1 | 3:1 | 3:1 | 3:1 |
| $\chi^{2}$ | 0.37 | 0.79 | 0.76 | 0.60 | 0.10 | 0.70 |
| *P* value | 0.54 | 0.37 | 0.38 | 0.44 | 0.75 | 0.40 |

“OE” represents the overexpression *BnaC04.BIL1* transformed lines.

**Table S5** Primers used in this study
